# Rationale for Early Administration of PCSK9 Inhibitors in Acute Coronary Syndrome

**DOI:** 10.31083/j.rcm2510374

**Published:** 2024-10-22

**Authors:** Salvatore Giordano, Jessica Ielapi, Nadia Salerno, Angelica Cersosimo, Alessandro Lucchino, Alessandro Laschera, Giovanni Canino, Assunta Di Costanzo, Salvatore De Rosa, Daniele Torella, Sabato Sorrentino

**Affiliations:** ^1^Department of Medical and Surgical Sciences, Division of Cardiology, “Magna Graecia" University, 88100 Catanzaro, Italy; ^2^Department of Experimental and Clinical Medicine, “Magna Graecia" University, 88100 Catanzaro, Italy

**Keywords:** acute coronary syndrome, proprotein convertase subtilisin/kexin type 9, low-density lipoprotein cholesterol

## Abstract

Acute coronary syndromes (ACSs) represent a significant global health challenge arising from atherosclerotic cardiovascular disease (ASCVD), with elevated low-density lipoprotein cholesterol (LDL-C) levels being a primary contributor. Despite standard statin therapy, individuals with ACS remain at high risk for recurrent cardiovascular events, particularly in the initial post-ACS period. Monoclonal antibodies targeting proprotein convertase subtilisin/kexin type 9 (PCSK9), such as evolocumab and alirocumab, offer a potential strategy to reduce LDL-C levels further and mitigate this residual risk. This review delves into the molecular mechanisms, effects on cholesterol metabolism, inflammatory modulation, and clinical outcomes associated with early administration of PCSK9 inhibitors following ACS.

## 1. Introduction

Acute coronary syndromes (ACSs) are an expression of atherosclerotic cardiovascular disease (ASCVD), ASCVD is one of the most prevalent causes of morbidity and mortality worldwide [1, 2].

Low-density lipoprotein cholesterol (LDL-C) is crucial for developing and progressing ASCVD and correlates with the risk of cardiovascular events [3, 4, 5]. Therefore, lowering LDL-C levels represents the central goal of the recent European Society of Cardiology/European Atherosclerosis Society and American Heart Association/American College of Cardiology guidelines for cardiovascular prevention [6, 7].

In patients who have suffered from an ACS, early plaque stabilization through LDL-C reduction is important for preventing recurrent ischemic events, as demonstrated by the substantial risk reduction of cardiovascular events among patients receiving an early and intense statin therapy [8, 9]. Notably, LDL-C targets vary according to the cardiovascular (CV) risk of the patient, with patients at very high risk requiring an LDL-C reduction ≥50%, with respect to the baseline level, and an LDL-C target below 55 mg/dL (1.4 mmL/L) [7, 10]. Nevertheless, despite current efforts, patients who have had an ACS remain at high risk for recurrent ischemic cardiovascular events [11, 12], especially within 1–3 months after the index event [13].

Evolocumab and alirocumab are two humanized monoclonal antibodies (mAbs) that target and inhibit the protein responsible for the catabolism of the LDL-C receptor: proprotein convertase subtilisin/kexin type 9 (PCSK9) [14]. These drugs significantly reduce LDL-C levels and are currently indicated in primary and secondary prevention of ASCVD for patients with persistently elevated LDL-C levels despite high-intensity statin therapy and in cases of statin intolerance [15].

Recently, some studies have tested the use of PCSK9 inhibitors (PCSK9I) in the acute phase after ACS for an early reduction in LDL-C, demonstrating the safety, feasibility, and efficacy of this approach [16, 17, 18]. Furthermore, it has been shown that using these drugs in the first days after ACS allows remodeling of the atherosclerotic plaque, possibly stabilizing the non-culprit lesions [19].

Early management of dyslipidemia using PCSK9I mAbs in the acute phase of ACS patients may improve outcomes in this context, and this review aims to underline the molecular mechanisms and current clinical evidence of this strategy.

## 2. Role of Proprotein Convertase Subtilisin/Kexin Type 9 in LDL-C Metabolism and Atherosclerosis

The PCSK9 enzyme, encoded by the *PCSK9* gene situated on chromosome 1 in humans, plays a key role in the degradation of LDL receptors (LDL-R) [20, 21]. Formerly known as NARC-1 (neural apoptosis-regulated convertase-1), this protein consists of 692 amino acids with a molecular weight of 72 kDa and was first elucidated in 2003 within the cerebral tissues of individuals afflicted with familial hypercholesterolemia [22]. Upon LDL binding to its receptor, the resultant complex undergoes internalization into hepatic cells within vesicles, subsequently merging with lysosomes for LDL degradation, while the receptor is recycled to the cell surface [20, 21]. However, the presence of PCSK9 in the LDL+ receptor complex instigates the degradation of LDL-R within the lysosomes, thus attenuating the number of receptors on the hepatic cell membrane, consequently impairing LDL clearance [20, 21].

PCSK9 circulates in plasma with a wide range of concentrations (33–2988 ng/mL) in healthy individuals, with its levels rising in response to conditions such as hypoxia, inflammatory stimulation, hemodynamic shear stress, or exposure to reactive oxygen species (ROS) [23]. While the liver, kidney, and small intestine are the main sources of circulating PCSK9, it is also produced by vascular cells, including vascular smooth muscle cells (VSMCs), endothelial cells (ECs), and, to a lesser extent, macrophages.

The contribution of PCSK9 to the atherosclerotic process goes beyond regulating LDL clearance, as it is directly involved in the process of vascular damage. A study by Tang *et al*. [24] in apolipoprotein E knockout (apoE KO) mice demonstrated that PCSK9 protein silencing led to less atherosclerotic burden than in the controls. Notably, the lesions in the PCSK9-depleted group had a reduced number of macrophages and decreased expression of vascular inflammation regulators, with a downregulation in the toll-like receptor 4 and nuclear factor kappa B (NF-κB) pathways [24]. Moreover, in macrophages, PCSK9 protein has been shown to upregulate gene expression and protein production of scavenger receptor (SR) A, cluster of differentiation 36 (CD36), and lectin-like oxidized low-density lipoprotein receptor 1 (LOX-1), which are involved in macrophage LDL uptake and oxidized LDL (ox LDL) formation [25, 26]. Finally, PCSK9 is also involved in the endothelial apoptosis process, further reducing vessel stability [27].

## 3. Molecular Rationale for Early Use of PCSK9 Inhibitors in Patients Post-Acute Coronary Syndrome

In the early phase of ACS, patients experience a surge in PCSK9 levels, which is partially linked to the high prescription of statins in this context [28, 29]. Indeed, statins reduce intracellular cholesterol reservoirs by inhibiting its endogenous production, which, in turn, stimulates PCSK9 transcription factors (sterol regulatory element binding proteins [SREBP] and hepatocyte nuclear factor 1α [HNF1α]), increasing circulating LDL levels [29]. This feedback mechanism might significantly elucidate the resistance observed in statin escalation strategies. Specifically, on average, with each doubling of statin dosage, there is a further 6% reduction in LDL-C levels [30].

Interestingly, several studies have also shown that a statin-independent mechanism contributes to the PCSK9 elevation in ACS. An analysis from the Ottawa Heart Genomics (OHGS) registry involving 45 individuals with acute myocardial infarction (AMI) revealed significantly elevated PCSK9 levels before initiating statin compared to 398 coronary artery disease (CAD) cases without myocardial infarction (MI). Similarly, in the Emory Cardiology Biobank study, PCSK9 levels were elevated in 74 individuals with AMI compared to the 273 individuals with CAD but no MI [31].

**Fig. 1.**
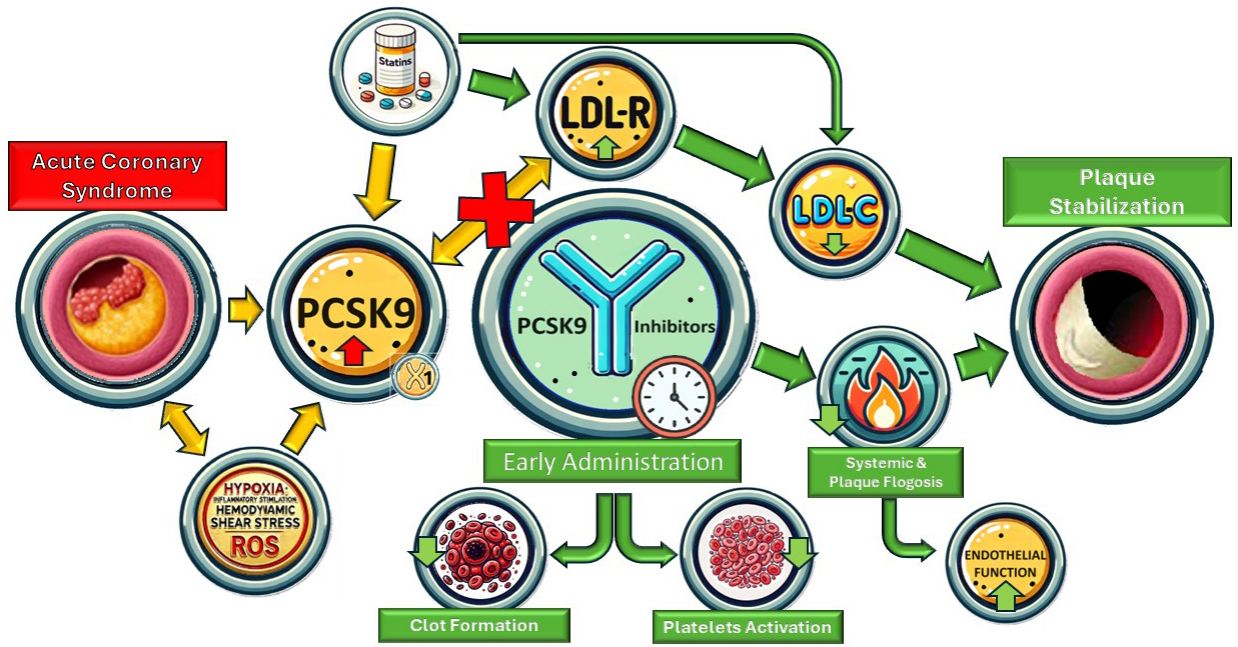
**Summary figure**. LDL-R, low-density lipoprotein receptor; PCSK9, proprotein convertase subtilisin/kexin type 9; LDL-C, low-density lipoprotein cholesterol; ROS, reactive oxygen species.

The molecular mechanism behind the surge in PCSK9 in patients who suffered from an ACS is unclear. However, evidence suggests that PCSK9 is associated with many pro-atherothrombotic states, including pro-inflammatory, pro-thrombotic, and endothelial pro-apoptotic states (Fig. 1).

### 3.1 PCSK9 and Inflammation

Evidence suggests that activating the inflammatory process during ACS could contribute to the increase in PCSK9 levels. The PEACE-Prospective AMI study revealed a positive correlation between plasma PCSK9 levels and high-sensitivity C-reactive protein (hs-CRP) levels, commonly used to assess systemic and low-grade inflammation about cardiovascular risk [32]. Likewise, within a substantial cohort of 2030 ACS patients undergoing coronary angiography in a prospective Swiss study, PCSK9 levels increased 12–24 hours after ACS presentation (374 + 149 vs. 323 + 134 ng/mL, *p* < 0.001). Moreover, patients with elevated PCSK9 levels during angiography demonstrated higher CRP levels [33]. Notably, this evidence is supported by studies conducted on mice, which have shown that lipopolysaccharides (LPS)-induced systemic inflammation increases hepatic PCSK9 mRNA levels by 12.5-fold [34].

Despite the correlation between PCSK9 and hs-CRP levels, a meta-analysis revealed that PCSK9Is have no significant effect on hs-CRP and, hence, no significant effect on systemic inflammation [35]. Similarly, secondary analyses from the EVOCATION trial (Evolocumab for prevention of microvascular dysfunction in patients undergoing percutaneous coronary intervention: the randomized, open-label EVOCATION trial), which was designed to assess the potential of PCSK9Is to reduce periprocedural microvascular resistance when administered six weeks before percutaneous coronary intervention (PCI), described no impact of PCSK9Is on hs-CRP, interleukin (IL)-6, and pentraxin-3 levels [36]. Overall, the interaction between systemic inflammation and PCSK9 remains elusive.

Even if PCSK9Is have failed to affect systemic inflammation markers significantly, recent data suggest they have an anti-inflammatory effect within atherosclerotic plaques. Hoogeveen *et al*. [37] demonstrated that using alirocumab for 14 weeks significantly reduced arterial wall inflammation, assessed by positron emission tomography/computed tomography (PET/CT), in 50 patients affected by CAD compared to controls, despite no changes in systemic inflammatory markers. Conversely, Marfella *et al*. [38] demonstrated, in a large translational study involving 645 patients who underwent carotid endarterectomy, that the use of PCSK9Is significantly reduces intraplaque expression of NLR family pyrin domain containing 3 (NLRP3) inflammasome and caspase-1 proteins, as well as lowers levels of IL-1β, tumor necrosis factor α (TNF-α), and NF-κB proteins in PCSK9Is-treated patients, compared to patients taking other lipid-lowering agents.

### 3.2 PCSK9 and Cardiac Injury

Laboratory investigations have demonstrated the constitutive expression of PCSK9 in adult terminally differentiated rat cardiomyocytes [39, 40]. Notably, its expression escalates under conditions of hypoxia, oxidized LDL exposure, hypoxia/reoxygenation, and ischemia/reperfusion scenarios [39, 41]. Specifically, exposure to hypoxia induces a significant upsurge in PCSK9 expression within cardiomyocytes, a phenomenon mitigated by HIF-1α siRNA intervention [39]. Moreover, PCSK9 inhibition confers protection against myocardial ischemia-reperfusion injury by suppressing autophagy pathways [39, 42]. Furthermore, a comprehensive study published in 2023 by Zhang and colleagues [43] demonstrated elevated PCSK9 expression in endothelial cells (ECs) under hypoxic conditions and in patients with critical limb ischemia compared to healthy individuals. Moreover, PCSK9 levels were higher in distal hypoxic vascular endothelium than in proximal normal endothelium. *In vitro* experiments showed that PCSK9 overexpression inhibited EC proliferation, migration, adhesion, and angiogenesis, whereas, in mice models, PCSK9 overexpression improved blood flow in ischemic limbs, while PCSK9 knockout reduced inflammatory factor release and enhanced blood flow [43].

### 3.3 Effect of PCSK9I on the Endothelium

PCSK9 is expressed in the artery wall, particularly in endothelium cells, smooth muscle cells, and macrophages [44]. The endothelium acts as a selectively permeable barrier. However, shear stress on the artery wall alters cell shape and orientation, increasing macromolecule permeability, such as LDL, promoting PCSK9 production, and activating inflammatory pathways [45]. Endothelial dysfunction, which disrupts the balance between vasodilator and vasoconstrictor factors, is the initial step in the atherosclerotic process [46]. PCSK9Is improve CV outcomes by directly reducing LDL-C levels and impacting systemic inflammatory responses, oxidative stress on the artery wall, and nitric oxide production [47].

Several studies have demonstrated that PCSK9Is are associated with improvements in arterial stiffness concurrent with LDL-C reduction [48], potentially enhancing endothelial function. Maulucci *et al*. [49] demonstrated proportional improvement in endothelial function with decreased LDL-C levels. For instance, in patients with a previous MI that was treated with evolocumab (140 mg bi-weekly) alongside maximum tolerated statin and ezetimibe doses, the authors observed improved flow-mediated dilation (FMD) following the brachial artery vasoreactivity test after two months of treatment [49]. Similar results were found by Di Minno *et al*. [50] in familial hypercholesterolemia (FH) patients treated with evolocumab (140 mg bi-weekly), whereby a reduction in small dense LDL and changes in oxidation markers and endothelial function were observed.

In patients with AMI, results from a sub-study of the PACMAN-AMI trial suggest improvement in endothelial dysfunction one year after the acute event [51]. However, there was no additional direct improvement in FMD with alirocumab compared to rosuvastatin alone and no significant association between LDL-C reduction and FMD improvement in the PCSK9I group [51]. These findings underscore the need for further research on the effects of PCSK9Is on endothelial function across different patient populations and possible differences during the acute phase of AMI.

### 3.4 PCSK9 and Platelet Reactivity

Elevated levels of PCSK9 have been correlated with heightened platelet reactivity and increased risk of ischemic major adverse cardiovascular events (MACEs). Laboratory studies support this hypothesis: the addition of hrPCSK9 to human-platelet-rich plasma samples significantly enhanced platelet aggregation induced by subthreshold concentrations of epinephrine (0.3 and 0.6 mM), reducing the lag time by 40% and increasing the area under the curve by 15% [52]. Furthermore, studies in mice have demonstrated that the absence of PCSK9 results in reduced arterial thrombus formation and stability, along with decreased platelet function. This evidence suggests that elevated PCSK9 levels may influence platelet reactivity, potentially predicting ischemic events in ACS patients undergoing PCI [52, 53, 54].

The effect of PCSK9 on platelet reactivity may be mediated by the CD36 receptor, which activates several pathways, such as Src kinase, mitogen-activated protein kinase (MAPK)- extracellular signal-regulated kinases (ERK)5, and c-Jun N-terminal kinases (JNK). These cascades increase the generation of ROS and thromboxane A2, ultimately intensifying platelet aggregability. In support of this hypothesis, Navarese *et al*. [55], in a recent analysis of 333 consecutive ACS patients treated with prasugrel or ticagrelor, showed a correlation between increased PCSK9 serum levels and platelet reactivity (r = 0.30; *p* = 0.004).

Notwithstanding these data, a mechanistic study conducted by Franchi *et al*. [56] has currently discarded the hypothesis of a significant impact of PCSK9 on platelet reactivity. In their study, Franchi *et al*. [56] examined the impact of evolocumab 420 mg on the pharmacodynamic profiles of clopidogrel in 84 ASCVD patients treated with clopidogrel. These patients were stratified into high platelet reactivity (HPR) and normal platelet reactivity (NPR) cohorts. The primary endpoint was P2Y12 reaction units (PRUs), assessed by Verify Now at 30 days. The study found that evolocumab significantly reduced LDL-C compared to the placebo at 14 and 30 days. At 14 days, the PRU levels were significantly lower in the evolocumab group compared to the placebo in the HPR cohort but not in the NPR cohort. At 30 days, there were no significant differences in PRUs in the HPR or NPR cohorts [56, 57].

### 3.5 PCSK9 and Coagulation Process

Several studies have demonstrated that PCSK9 exhibits direct thrombotic and hypercoagulative activities, which promote the atherosclerotic process [47, 58]. Therefore, by providing antithrombotic activity, administering PCSK9Is may reduce CV events. Zhang *et al*. [59] showed that PCSK9 levels were positively associated with circulating fibrinogen in patients with stable CVD. Furthermore, by lowering the LDLR-related protein (LRP) levels, PCSK9 increases the plasma concentration of factor VIII (FVIII), which represents a critical element in the coagulation cascade [60].

The effects of PCSK9 on fibrinolysis are also linked to its correlation with plasminogen activator inhibitor-1 (PAI-1), which promotes the thrombotic process by inhibiting the tissue-type plasminogen activator (t-PA) and urokinase-type plasminogen activator (uPA) [61]. Levine *et al*. [61] demonstrated a direct correlation between PAI-1 levels and PCSK9 concentrations through RNA sequencing *in vitro* and *in vivo* in mice with hyperlipidemia. Additionally, the authors documented a positive correlation between PAI-1 and PCSK9 levels in patients with heart failure and preserved ejection fraction. After treatment with PCSK9Is in hypercholesterolemic patients, a statistically significant increase in PCSK9 levels and reduction in PAI-1 concentrations were observed, suggesting that PCSK9 modulation has varying effects on PAI-1 levels [61].

Basiak *et al*. [58] demonstrated that alirocumab (150 mg bi-weekly) used for 90 days in patients with isolated hypercholesterolemia is associated with significatively reduced PAI-1, factor VII, and fibrinogen, compared to the baseline and control group, and reduced von Willebrand factor (vWF) levels compared to the baseline.

Likozar *et al*. [62] randomized 100 stable post-MI patients with uncontrolled LDL-C levels and elevated Lp(a) to receive either placebo or a PCSK9I (alirocumab [150 mg bi-weekly] or evolocumab [140 mg bi-weekly]). All the groups showed significantly increased PAI-1 levels and a borderline increase in thrombin activatable fibrinolysis inhibitor (TAFI) levels in patients treated with PCSK9Is (*p* = 0.062). Various factors, including previous statin treatment for all patients in the study, could influence the PAI-1 concentration, explaining the increased fibrinolytic parameter. However, the results highlighted the correlation between LDL-C concentrations and PAI-1 (*p* = 0.049), as well as TAFI (*p* < 0.001) in the early phase post-MI. This correlation disappeared after six months of lipid-lowering treatments [62].

Overall, these findings suggest that PCSK9 may influence both primary and secondary hemostasis, either indirectly through its impact on LDL-C levels or directly by affecting hemostatic parameters. PCSK9Is may exhibit a multifaceted effect on fibrinolysis and coagulation, thereby suggesting potential future benefits.

## 4. Benefits of Early PCSK9I on Plaque Composition

Numerous studies utilizing intracoronary optical coherence tomography (OCT) have identified hallmark features of vulnerable plaque associated with adverse cardiovascular outcomes [63, 64]. They include the presence of a thin fibrous cap, large lipid pool, cholesterol crystals, spotty calcification, neovascularization, and potentially macrophage collections in culprit lesions and other sites within the vasculature of ACS patients [63, 64].

The phase 3, multicenter, double-blind HUYGENS (High-Resolution Assessment of Coronary Plaques in a Global Evolocumab Randomized Study) [63] evaluated the effect of evolocumab on plaque composition via serial OCT measures in patients who suffered from an non-ST elevation myocardial infarction (NSTEMI). The lesions evaluated in this trial were non-culprit plaques, which determined an angiographic stenosis of ≥20%. They had to have at least 1 OCT image with a fibrous cap thickness (FCT) ≤120 µm and one with a lipid arc >90° in a segment at least 40 mm long. A total of 161 patients were enrolled and randomized to evolocumab 420 mg or placebo, on top of statin therapy, once a month for 52 weeks. The primary endpoint was the nominal change in minimum FCT at 50 weeks, which was greater in the evolocumab group vs. placebo (+42.7 vs. +21.5 µm; *p* = 0.015). Moreover, the evolocumab group showed a decrease in the maximum lipid arc (–57.5° vs. –31.4°; *p* = 0.04) and macrophage index (–3.17 vs. –1.45 mm; *p* = 0.04) throughout the arterial segment, compared to the placebo group [63].

The PACMAN-AMI trial (Effects of the PCSK9 Antibody Alirocumab on Coronary Atherosclerosis in Patients with Acute Myocardial Infarction) showed a similar effect of alirocumab on plaque composition. This is a double-blind, placebo-controlled, randomized clinical trial that enrolled 300 patients who suffered from MI [64]. Patients, in addition to statin therapy, were randomized to receive biweekly subcutaneous alirocumab (150 mg; n = 148) or placebo (n = 152), starting from less than 24 hours from urgent PCI of the culprit lesion, for 52 weeks. The study was powered for three endpoints: change in percent atheroma volume (PAV) (primary endpoint, via intravascular ultrasound (IVUS)), change in maximum lipid core burden index within 4 mm (secondary endpoint, via near-infrared spectroscopy (NIRS)), and change in minimal FCT (secondary endpoint, via OCT). The results illustrated a greater reduction in the mean PAV from baseline in the alirocumab group, compared with the placebo group (–2.13% [95% CI, –2.53% to –1.73%] vs. –0.92% [95% CI, –1.28% to –0.56%]; between-group difference, –1.21% [95% CI, –1.78% to –0.65%]; *p* < 0.001). Moreover, a significantly greater reduction in maximum lipid core burden index within 4 mm was found in the alirocumab group vs. the placebo group (–79.42 vs. –37.60; between-group difference, –41.24 [95% CI, –70.71 to –11.77]; *p* = 0.006). Lastly, a greater increase in mean minimal FCT was shown in the alirocumab group (62.67 µm [95% CI, 48.84–76.50]) compared with the placebo group (33.19 µm [95% CI, 22.22–44.16]) (between-group difference, 29.65 µm [95% CI, 11.75–47.55]); *p* = 0.001) [64].

In addition to stabilizing vulnerable plaques, long-term use of PCSK9Is has been shown to reduce plaque burden compared to intensive statin treatment, with a degree of benefit proportional to the extent of LDL-C lowering achieved. This evidence comes from the GLAGOV (Global Assessment of Plaque Regression with a PCSK9 Antibody as Measured by Intravascular Ultrasound) trial [65]. This double-blind, randomized controlled trial included patients on a stable dose of statins, with coronary stenosis >20% and LDL-C >80 mg/dL or between 60 and 80 mg/dL plus additional cardiovascular risk factors, who were randomized to evolocumab 420 mg/month vs. placebo, for 76 weeks. The primary endpoint was the reduction in percent atheroma volume, evaluated by IVUS, which showed a significant decrease by 0.95% in the evolocumab group and no difference in the placebo group, with a between-group difference of –1.0% [95% CI, –1.8% to –0.64%] (*p* < 0.001) [65].

Overall, adding PCSK9Is to statin therapy could lead to an additional increase in fibrous cap thickness and reductions in lipid-rich plaque, even in the short term during the initial stages following ACS. However, the molecular mechanism behind this process has yet to be fully understood. A study conducted by Basiak *et al*. [66] in patients treated with PCSK9Is suggests that the therapy modulates the levels of pro-atherogenic cytokines, including osteopontin, osteoprotegerin, and metalloproteinase 9, which may contribute to the process of plaque stabilization. Moreover, prolonged administration of PCSK9Is may delay plaque progression and reduce plaque burden.

## 5. Impact of Early Use of PCSK9Is on Lipids and Clinical Outcomes

The impact of the early use of PCSK9Is after ACS on clinical outcomes has never been investigated in large clinical trials. The largest phase 3 studies on PCSK9Is excluded patients who suffered from an MI <4 weeks (i.e., the FOURIER (further cardiovascular outcomes research with PCK9I in patients with elevated risk) [67] and the ODYSSEY outcomes (evaluation of cardiovascular outcomes after an acute coronary syndrome during treatment with alirocumab) [68]). Nevertheless, smaller studies enrolling patients admitted for an ACS assessed the effect of an early prescription of PCSK9Is on lipids (Table 1, Ref. [16, 17, 63, 64, 65, 69, 70, 71, 72, 73, 74]).

**Table 1.** **Lipid profile characteristics across studies after treatment with PCSK9I or placebo**.

Study	Follow-up	Values (mg/dL)	Randomization		*p*-value
Trankle *et al*. 2019 [16]	14 days		Alirocumab 150 mg once	Placebo	
(VCU-AlirocRT)			(Pts = 10)	(Pts = 10)	
		LDL	28 (14–51)	90 (75–131)	<0.001
Nicholls *et al*. 2022 [63]	350 days		Evolocumab 420 mg/mo	Placebo	
(HUYGENS)			(Pts = 80)	(Pts = 81)	
		LDL	28.1 ± 25.4	87.2 ± 36.5	<0.001
		HDL	51.2 ± 13.2	47.1 ± 12.4	0.26
		TG	114.8 ± 84.9	133.5 ± 57.5	0.13
Okada *et al*. 2020 [72]	28 days		Evolocumab 140 mg bi/weekly	Placebo	
			(Pts = 51)	(Pts = 49)	
		LDL	–92.4 ± 32.4^*^	−44.8 ± 32.1^*^	<0.001
Mehta *et al*. 2022 [69]	42 days		Alirocumab 150 mg bi-weekly	Placebo	
(EPIC STEMI)			(Pts = 38)	(Pts = 30)	
		LDL	29 ± 17.8	50.3 ± 17.4	<0.001
		HDL	42.2 (35.6–47.6)	37.9 (33.3–47.6)	0.29
		TG	89.4 (72.6–123.9)	105.3 (77.9–143.4)	0.044
Hao *et al*. 2022 [71]	30 days		Evolocumab 140 mg bi-weekly	Placebo	
			(Pts = 68)	(Pts = 68)	
		LDL	22.04 ± 17.4	48.7 ± 18.9	<0.01
		HDL	43.7 ± 10.1	41.8 ± 8.5	0.269
		TG	96.5 ± 51.3	93.8 ± 45.2	0.749
Wang *et al*. 2022 [73]	30 days		Evolocumab 140 mg once	Placebo	
			(Pts = 35)	(Pts = 30)	
		LDL	56.1 ± 15.1	77.7 ± 19.7	0.18
		HDL	36 ± 9.3	32.5 ± 8.5	0.32
		TG	130.12 ± 65.5	170.84 ± 88.5	0.19
Räber *et al*. 2022 [64]	365 days		Alirocumab 150 mg bi-weekly	Placebo	
(PACMAN-AMI)			(Pts = 126)	(Pts = 132)	
		LDL	23.6 ± 23.8	74.4 ± 30.5	<0.001
		HDL	48.3 ± 11.2	45.0 ± 11.6	<0.001
		TG	94.2 ± 47.0	126.0 ± 77.9	<0.001
Nicholls *et al*. 2016 [65]	546 days		Evolocumab 420 monthly	Placebo	
(GLAGOV)			(Pts = 484)	(Pts = 484)	
		LDL	36.6 (34.5–38.8)	93.0 (90.5–95.4)	<0.001
		HDL	51.0 (49.8–52.1)	47.1 (46.0–48.2)	<0.001
		TG	105.1 (82.5–141.6)	130.5 (100.3–177.2)	<0.001
Koskinas *et al*. 2019 [17]	56 days		Evolocumab 420 mg monthly	Placebo	
(EVOPACS)			(Pts = 155)	(Pts = 153)	
		LDL	30.5 ± 17.8	79.7 ± 24.4	<0.001
		HDL	46.4 ± 12.8	45.6 ± 12.8	0.56
		TG	117.8 ± 63.8	127.6 ± 65.5	0.25
Vavuranakis *et al*. 2022 [70]	30 days		Evolocumab 420 mg/mo	Placebo	
			(Pts = 39)	(Pts = 35)	
		LDL	34.7 ± 22.2	61.8 ± 24.7	<0.01
		HDL	45.4 ± 10.7	45.5 ± 16.8	0.97
		TG	87 (57–126)	85 (69–134)	0.55
Luo *et al*. 2023 [74]	30 days		PCSK9I monthly	Placebo	
			(Pts = 224)	(Pts = 259)	
		LDL	50.7 (33.3–64.2)	82.4 (66.2–102.2)	<0.01
		HDL	37.1 (32.4–44.3)	37.9 (32.5–44.1)	0.933
		TG	136 (95–175.4)	123.2 (93.9–174.5)	0.233

Values are mean ± SD or median (IQR). * Change in LDL-C from the baseline value. TG, triglycerides; HDL, high-density lipoprotein cholesterol; LDL, low-density lipoprotein cholesterol; PCSK9I, pro-protein convertase subtilisin/kexin type 9 inhibitor; SD, standard deviation; IQR, interquartile range.

The EVOPACS (evolocumab for early reduction of LDL in patients with acute coronary syndrome) study [17] is an investigator-initiated, randomized, double-blind, placebo-controlled trial including 308 patients hospitalized for ACS with elevated LDL-C levels. Enrolled patients were randomly assigned to receive either evolocumab 420 mg (n = 155) or placebo (n = 153) as early as possible (within ≤24 h), with the primary endpoint being the percentage change in calculated LDL-C from baseline to 8 weeks. Results demonstrated a significant reduction in LDL-C levels in the evolocumab group compared to placebo (77.1 ± 15.8% vs. 35.4 ± 26.6% in the placebo group; *p* < 0.001). At 8 weeks, a high proportion (95.7%) of patients in the evolocumab group obtained LDL-C <1.8 mmol/L, compared with only 37.6% of the patients in the placebo group. Evolocumab also significantly reduced other lipid parameters after 8 weeks, including total cholesterol (TC) by 26.5%, apolipoprotein B (Apo-B) by 34.2%, and non-HDL-C by 34.6%, with a mean increase in HDL-C of 4.8% [17]. Adverse events did not differ between groups after 8 weeks of follow-up. Most events involved coronary revascularization procedures, with a higher proportion, albeit non-significant, in the placebo group than in the evolocumab group (32 vs. 38 pts.). Target lesion revascularization was rare, and recurrent MI occurred in five patients, four of whom were in the evolocumab group, including two who died [17].

The EPIC STEMI (effects of routine early treatment with PCSK9 inhibitors in patients undergoing primary percutaneous coronary intervention for ST-segment elevation myocardial infarction) trial [69] is an investigator-initiated randomized, double-blind, placebo-controlled clinical study assessing the impact of alirocumab on lowering lipid levels in ACS ST- elevation MI (STEMI) patients undergoing PCI. Patients were randomized to alirocumab added to high-intensity statin therapy irrespective of the baseline LDL-C level versus sham control. The primary outcome was the percentage reduction in direct LDL-C up to 6 weeks. At a median 45 days follow-up, alirocumab (150 mg bi-weekly), administered before PCI, resulted in a significant reduction in LDL-C of 72.9% (from 2.97 mmol/L to 0.75 mmol/L) compared to a 48.1% reduction (from 2.87 mmol/L to 1.30 mmol/L) in the sham-control group at 45 days (*p* < 0.001) [69]. The between-group difference in LDL-C reduction was 22.3% (95% CI: –31.1 to –13.5; *p* < 0.001). Secondary outcomes also showed favorable decreases in the levels of Apo-B (50.6% vs. 36.3%; between-group difference of 11.5%, *p* < 0.001) and non-HDL-C (between-group difference 19.1%, *p* = 0.001), along with a significant difference in median lipoprotein (a) (Lp(a)) levels, demonstrating alirocumab’s inhibitory effect (alirocumab +5.3%, interquartile range (IQR) 0 to 40.2 vs. sham control +34.8%, IQR 14.7 to 68.6; *p* = 0.023). Clinical events were rare in this study. Among the 97 patients enrolled, there was one death in the sham-control group and none in the alirocumab group. No myocardial infarctions or strokes occurred in either group [69].

The EVACS (Evolocumab in Acute Coronary Syndrome; ClinicalTrials.gov, Unique identifier: NCT03515304) trial [18] enrolled 57 patients with non-ST-segment–elevation myocardial infarction and troponin I of ≥5 ng/mL and randomized them, on top to a high-intensity statin regimen, to a single dose of evolocumab or matching placebo within 24 hours of presentation. Evolocumab promoted a significant reduction in LDL-C levels (primary outcome) from baseline by 28.4 ± 4 mg/dL (*p* < 0.0001), evident as early as day 1 (70.4 ± 27 mg/dL; *p* < 0.01 vs. baseline), which was sustained throughout hospitalization and at the 30-day follow-up (*p* < 0.01) [18]. Linear regression analysis, adjusted for baseline LDL-C, statin use, change in statin, and ezetimibe use, indicated an average LDL-C reduction of 28.6 mg/dL, lower than in the evolocumab group compared to the placebo group at 30 days (*p* < 0.0001). Additionally, non–HDL-C and Apo-B levels were significantly lower in the evolocumab group at hospital discharge and 30 days. No significant differences were observed in triglycerides or HDL-C levels [18].

A pooled analysis [70] of patients enrolled in the EVACS I and in the EVACS II trial (still ongoing), including 74 patients with an ACS diagnosis, who were randomized to receive evolocumab or placebo within 24 hours of hospitalization, revealed that the early use of PCSK9Is is not associated to a surge in Lp(a) levels at 30 days, which occurred in the placebo group. In fact, at 30 days, the placebo group showed a significant increase in Lp(a) to 82 nmol/L (24% median percentage increase, *p* < 0.01), while the evolocumab group exhibited no significant change (44 nmol/L, 0% median percentage change, *p* = 0.86) [70].

In the Virginia Commonwealth University—Alirocumab Rapid Thrombus Resolution (VCU-AlirocRT) trial [16], patients with NSTEMI, already receiving high-intensity statin therapy, with LDL-C ≥70 mg/dL in the past 12 months, were enrolled. A total of 20 patients were randomly assigned in a 1:1 ratio to receive alirocumab 150 mg or placebo, with immediate administration. The primary endpoint was a placebo-corrected change in LDL-C at 14 days. Baseline characteristics were similar between groups, except for lower Lp(a) levels in the placebo group (56 vs. 215 nmol/L, *p* = 0.043). During the trial, LDL-C levels in the placebo group remained unchanged [98 (72–160) mg/dL baseline to 93 (61–132) and 90 (75–131) mg/dL at 72 hours and 14 days, *p* > 0.1]. Conversely, the alirocumab group showed a significant reduction in LDL-C from 91 (71–129) mg/dL at baseline to 73 (36–110) at 72 hours (*p* = 0.02) and 28 (14–51) mg/dL at 14 days (nd *p* < 0.01). Adverse events were minimal: one in the placebo group (sepsis) and four in the alirocumab group (one stroke, two heart failure admissions, one unstable angina readmission), not attributed to the medication (*p* = 0.152). Interestingly, secondary inflammatory outcomes were assessed in the VCU-AlirocRT trial; however, no particular effect of evolocumab was shown. The hs-CRP levels remained stable throughout the study (*p* > 0.2 for all comparisons). Interleukin-6 levels decreased at day 14 (*p* < 0.05), but there were no between-group differences (*p* > 0.4). Tumor necrosis factor-α and interleukin-10 levels showed no significant changes [16].

The study by Hao *et al*. [71] in 2022 was a prospective, randomized, controlled trial involving 136 patients diagnosed with extremely high-risk ACS. Patients were randomly assigned to receive either evolocumab (within 48 hours after PCI) or the placebo, in addition to standard statin therapy. During the first month, the evolocumab group demonstrated a substantial reduction in LDL-C levels, decreasing by –83.88% ± 13.44%, whereas the control group exhibited a reduction of –63.89% ± 13.85% (*p* < 0.01). Furthermore, a significantly higher percentage of patients in the evolocumab group (82.35%) achieved the target LDL-C value (<1.0 mmol/L), compared to only 22.06% in the control group (*p* < 0.01). At three months, the evolocumab group maintained lower LDL-C levels (0.58 ± 0.26 mmol/L) in contrast to the control group (1.27 ± 0.54 mmol/L, *p* < 0.01). Additionally, there were more significant reductions in other lipid parameters (*p* < 0.01), including Apo B/A1, TC, and Apo-B, observed in the evolocumab group compared to the control group [71].

The study by Okada *et al*. in 2020 [72] was a single-center, randomized, controlled trial of 98 patients hospitalized for AMI designed to evaluate the feasibility and safety of early initiation of evolocumab in patients undergoing primary PCI. All patients also received pitavastatin (2 mg/day) and were randomized to evolocumab 140 mg or placebo within 24 hours post-PCI and every 2 weeks [72]. The primary outcome was a change in LDL-C levels from baseline to 4 weeks. LDL-C levels decreased significantly after 4 weeks, with a reduction of 76.1% in the evolocumab group compared to 33.1% in the control group (mean difference: –43.9%; 95% CI: –52.1 to –35.6%; *p* < 0.001). Achieving LDL-C levels <70 mg/dL at 4 weeks was observed in all patients in the evolocumab group and 27% in the control group. In the evolocumab group, non-HDL-C decreased by 66.2% vs. 26.0% in controls (*p* < 0.01). HDL-C changes were minimal (2.8% vs. –0.7%, *p* = 0.48), while small dense LDL decreased by 67.3% with evolocumab compared to 13.8% in controls (*p* = 0.01). Evolocumab led to a –2.7% change in Lp(a), contrasting with an 82.0% increase in controls (*p* = 0.01). Adverse events did not significantly differ between groups (17 vs. 21 total; 2 vs. 2 serious) [72].

Another small single-center, prospective, randomized, open-labeled trial published by Nakamura and colleagues in 2020 [75] enrolled 38 patients diagnosed with STEMI, of whom 17 received evolocumab, and 19 were in the non-evolocumab group. In the non-evolocumab group, LDL-C levels decreased significantly from day 0 to day 3, followed by a temporary increase at day 5. In contrast, the evolocumab group continuously decreased to 30.7 ± 18.9 mg/dL at day 10. Plasma Lp(a) levels in the non-evolocumab group peaked on day 3 and returned to baseline by day 10. Conversely, the evolocumab group experienced a significant decrease in plasma Lp(a) levels during AMI [75].

Secondary endpoints of the previously mentioned HUYGENS and PACMAN-AMI trial, which were designed to evaluate plaque remodeling, include changes in LDL-C at follow-up. In the HUYGENS [63], at 50 weeks of follow-up, LDL-C was significantly reduced from 140.4 to 28.1 mg/dL in the evolocumab group, compared to a decrease from 142.1 to 87.2 mg/dL in the placebo (between groups *p* < 0.001). In the PACMAN-AMI trial [64], at 52 weeks of follow-up, LDL-C levels were significantly higher in the control group, which were 74.4 ± 30.5 mg/dL, whereas in the alirocumab group, they were 23.6 ± 23.8 mg/dL (*p* < 0.001). Notably, in both trials, there were few adverse events. In the HUYGENS [63], the incidence of cardiovascular events was minimal, and there was no notable contrast in mortality rates (0% vs. 1.2%) or occurrences of myocardial infarction (0% vs. 3.7%) between patients treated with evolocumab and those administered the placebo. In the PACMAN-AMI trial [64], alirocumab had two cases (1.4%) of all-cause mortality, two cases (1.4%) of cardiac death, two cases (1.4%) of myocardial infarction, and 12 cases (8.2%) of ischemia-driven coronary revascularization. In contrast, the placebo group had 1 (0.7%), 0, 3 (2.0%), and 28 (18.5%) cases, respectively.

In a randomized study conducted in Shanxi Cardiovascular Disease Hospital in Taoyuan [73], a population of 65 patients with STEMI was divided into a routine pre-treatment group receiving high-intensity statin alone (40 mg atorvastatin or 20 mg rosuvastatin) and a combined treatment group receiving high-intensity statin plus PCSK9I (injection of evolocumab 140 mg). The primary endpoint was represented by the evaluation of the myocardial perfusion with a series of parameters, and between these, the corrected thrombolysis in myocardial infarction (TIMI) frame count (CTFC) was significantly lower, and TIMI myocardial perfusion grading (TMPG) was significantly improved both immediately after revascularization and in coronary angiography after one month. Nevertheless, combined treatment did not decrease the incidence of cardiovascular death, non-fatal MI, or target vessel revascularization, although, in the routine treatment group, there were two cases of plaque progression [73].

In a Chinese retrospective study [74], 483 patients were divided into a PCSK9I group (n = 224) and a control group (n = 259). The PCSK9I group received statins and short-term PCSK9I for 3 months, while the control group received only statins. Composite endpoint events included cardiac death, recurrent MI, stroke, unstable angina, heart failure hospitalization, and revascularization. Despite the PCSK9I group having a lower mean age (*p* < 0.01) and higher body mass index (BMI) (*p* < 0.01), they achieved significantly higher rates of LDL-C target goals at 1-month post-discharge compared to the control group (*p* < 0.01). However, LDL-C target rates at 1-year were low and did not differ significantly between groups. Composite endpoint event incidence did not significantly differ between groups at 1 month (*p* = 0.865), but at 1-year, it was significantly lower in the PCSK9I group (*p* = 0.013), mainly due to fewer recurrent unstable angina events (*p* = 0.029). This suggests that achieving LDL-C targets shortly after ACS could significantly affect long-term cardiovascular outcomes. Short-term use of PCSK9Is for 3 months, initiated early, significantly reduced 1-year composite endpoint events in ACS patients, primarily by decreasing recurrent unstable angina events [74].

It is necessary to underscore that the majority of the studies discussed above included patients already receiving statin therapy, while the benefits of PCSK9Is on clinical outcomes may differ for patients who are statin-intolerant and not undergoing statin therapy. For this reason, it would be important to conduct large-scale studies that include more patients not receiving statin treatment to validate PCSK9I benefits in this scenario. Similar information could be gathered from studies on Inclisiran, a small interfering RNA (siRNA) that suppresses the expression of PCSK9 messenger RNA (mRNA) in the liver. However, studies on clinical outcomes remain limited [14].

## 6. Conclusions

Despite intensive statin therapy, ACS patients remain at high risk for recurrent ischemic events, especially within the initial months. Beyond modulating LDL levels, PCSK9 is involved in multiple pathological processes that contribute to the development of ACS, including inflammation and thrombosis. Thus, prompt use of PCSK9 inhibitors may prevent early- ACS recurrences. These hypotheses are supported by the fast-occurring plaque-stabilizing effect associated with the use of these drugs. However, future studies with appropriate designs to test these hypotheses are needed.
